# Endoscopic Injection Sclerotherapy With Pre‐deflation Ligation for Prolonged Sclerosant Retention in Esophageal Varices: A Case Report

**DOI:** 10.1002/deo2.70410

**Published:** 2026-09-03

**Authors:** Noriaki Sugawara, Taro Iwatsubo, Masatoshi Kaizuka, Sayaka Tsuji, Katsutoshi Nishian, Kazuki Takayama, Shun Sasaki, Akitoshi Hakoda, Kazuhiro Ota, Hiroki Nishikawa

**Affiliations:** ^1^ Second Department of Internal Medicine Osaka Medical and Pharmaceutical University Osaka Japan

**Keywords:** endoscopy, esophageal and gastric varices, ethanolamine oleate, ligation, sclerotherapy

## Abstract

Esophageal varices are a common complication of portal hypertension, and prophylactic endoscopic injection sclerotherapy with ligation (EISL) is an established treatment option. Effective variceal thrombosis requires sufficient intravariceal retention of the sclerosant, because ethanolamine oleate exerts its thrombogenic effect through prolonged contact with the venous endothelium. In conventional EISL, endoscopic variceal ligation (EVL) is performed after balloon deflation, leaving a transient interval during which restored blood flow and an open puncture site may permit washout of the sclerosant and bleeding. We describe EIS with pre‐deflation ligation (EISpdL), in which EVL of the puncture site is completed while the endoscope balloon remains inflated, and balloon compression is maintained throughout the retention period. A 59‐year‐old man with medium‐sized esophageal varices with a positive red color sign (F2, RC1) secondary to metabolic dysfunction‐associated steatohepatitis underwent EISpdL combined with gel‐immersion EIS. The procedure was technically feasible, required no additional skills beyond conventional EISL, and no immediate procedure‐related adverse events were observed. Follow‐up endoscopy on post‐procedure day 7 showed bronze‐colored variceal changes indicating thrombosis, and contrast‐enhanced computed tomography two months later confirmed obliteration. EISpdL may enable more stable and prolonged sclerosant retention.

## 1 Introduction

Esophageal varices are a common complication of portal hypertension, and rupture can cause life‐threatening bleeding; prophylactic endoscopic treatment is therefore recommended for varices at high risk of rupture [1]. Endoscopic injection sclerotherapy with ligation (EISL) has been established as one of the treatment options for esophageal varices [1, 2]. Compared with conventional EIS, EISL has been reported to shorten the procedure time, reduce paravariceal leakage of the sclerosant, achieve higher intravascular injection success rates, particularly for small varices, and reduce post‐treatment adverse events [3]. Although the precise procedural sequence of EISL varies among institutions, in a common protocol an endoscope balloon is inflated to occlude oral‐side variceal flow, a sclerosant (5% ethanolamine oleate [EO]) is injected intravariceally, and, after balloon deflation, endoscopic variceal ligation (EVL) is applied to the puncture site for hemostasis.

Effective variceal thrombosis depends on adequate intravariceal retention of the sclerosant, because EO produces thrombosis through destruction of the venous endothelium, an effect that is dependent on sufficient endothelial exposure time [4]. However, maintaining stable intravariceal needle positioning during injection is often technically difficult because of body movement and esophageal peristalsis, and premature needle dislodgement before adequate retention may cause bleeding at the puncture site.

To address these limitations, we developed EIS with pre‐deflation ligation (EISpdL), in which EVL of the puncture site is completed before the balloon is deflated, so that sustained balloon compression maintains occlusion of the oral‐side variceal outflow throughout the retention period. Here, we report the first case treated with this technique.

## 2 Case Report

Screening upper gastrointestinal endoscopy in a 59‐year‐old man with cirrhosis secondary to metabolic dysfunction‐associated steatohepatitis revealed esophageal varices located in the middle and lower esophagus, classified as medium‐sized varices with a positive red color sign (Lm, F2, RC1 according to the Japanese classification of the Japan Society for Portal Hypertension), and he was admitted for prophylactic endoscopic treatment. Written informed consent for the procedure and for publication was obtained from the patient.

The procedure was performed by two board‐certified endoscopists who are members of the Japan Gastroenterological Endoscopy Society. A double‐type overtube (TOP Corporation, Tokyo, Japan) was then inserted, and the endoscope was withdrawn. A modified pneumo‐activated EVL device (Sumitomo Bakelite Co., Ltd., Tokyo, Japan) and a 6‐cm balloon for endoscopy (φ 11 × 60 mm; CREATE MEDIC Co., Ltd., Yokohama, Japan) were mounted on the tip of the endoscope, and the endoscope was reinserted. After degassing the esophagus and stomach, the attached balloon was inflated with air to occlude oral‐side variceal blood flow. The inflation volume is usually 20–30 mL and is adjusted as appropriate according to the fluoroscopic findings and the contrast findings during sclerosant injection; in the present case, the balloon was inflated with 30 mL of air.

**FIGURE 1:**
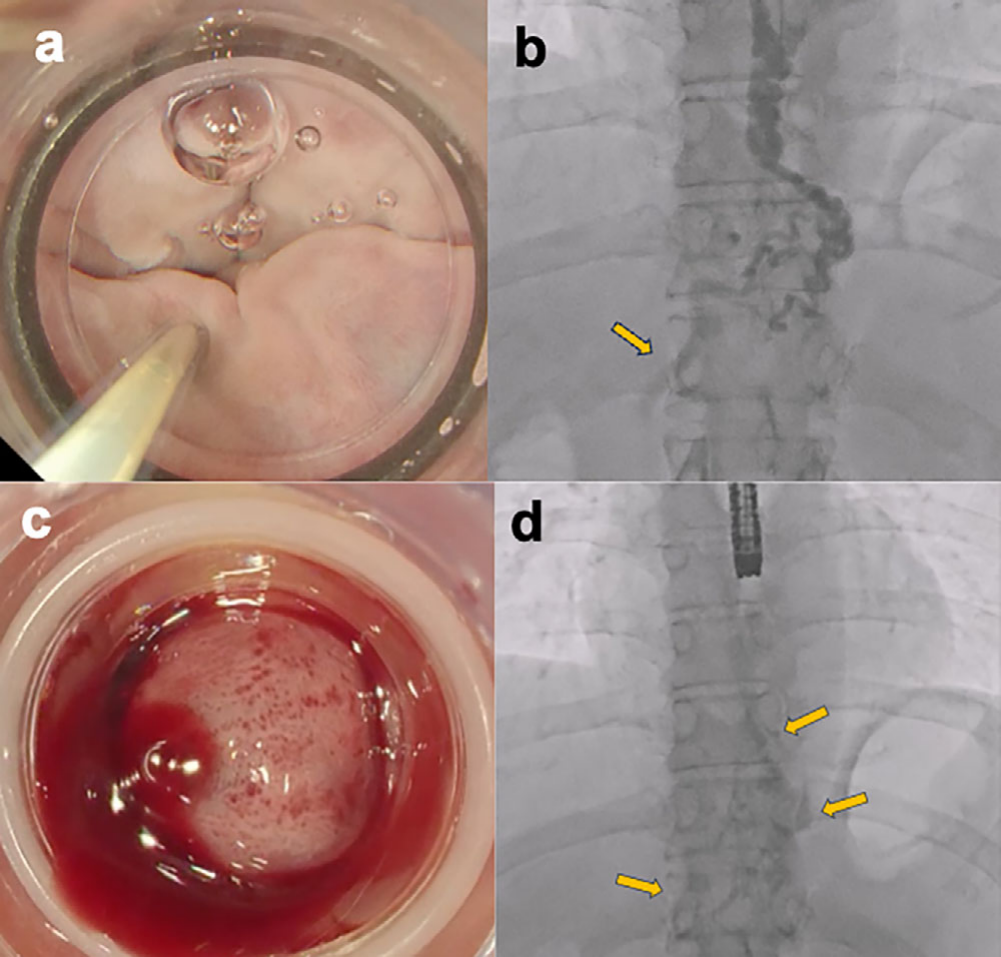
Endoscopic and fluoroscopic findings during EIS with pre‐deflation ligation (EISpdL). (a) Endoscopic image during gel‐immersion EIS; the varix, dilated under gel immersion, was punctured, blood backflow into the needle was confirmed, and the sclerosant was injected. (b) Fluoroscopic image showing retrograde opacification of the left gastric vein (LGV), the main afferent vessel; injection was terminated when the contrast medium reached the origin of the LGV (yellow arrow). (c) Endoscopic image showing the puncture site after needle withdrawal, followed by endoscopic variceal ligation (EVL). (d) Fluoroscopic image showing retention of the sclerosant within the LGV (yellow arrows); by maintaining balloon inflation during EVL, the sclerosant was retained within the varices.

Gel‐immersion EIS was then performed [5, 6, 7]. Gel was injected through the accessory channel to fill the esophageal lumen, and the varix was punctured under gel immersion using a 23‐gauge disposable puncture needle (Variceser type S; TOP Corporation). After confirming blood backflow, a mixture of 10% EO and a non‐ionic contrast agent (iopamidol) in a 1:1 ratio, yielding a final EO concentration of 5%, was injected retrogradely into the varix. Under fluoroscopy, the injection was terminated when the sclerosant reached the origin of the left gastric vein (LGV), the main afferent vessel (Figure 1a,b). A total of 20 mL of the mixture (5% EO) was injected.

With the balloon kept inflated, the needle was withdrawn, and the puncture site was immediately ligated by EVL (Figure 1c). Balloon compression of the oral‐side variceal segment was maintained throughout a 5‐min retention period, during which the sclerosant was retained within the varix and the LGV (Figure 1d; Video S1). The balloon was then deflated, which restored blood flow and washed out the residual sclerosant, and the endoscope was withdrawn. The total procedure time was approximately 14 min. Nearly the entire intended volume of EO was delivered before ligation. No immediate procedure‐related adverse events were observed in this case.

Follow‐up endoscopy on post‐procedure day 7 revealed bronze‐colored changes in all target varices, indicating successful thrombosis, and the patient was discharged without additional treatment. Contrast‐enhanced computed tomography two months later demonstrated no enhancement of the LGV or the esophageal varices, confirming obliteration. No variceal recurrence was observed during subsequent follow‐up.

## 3 Discussion

**FIGURE 2:**
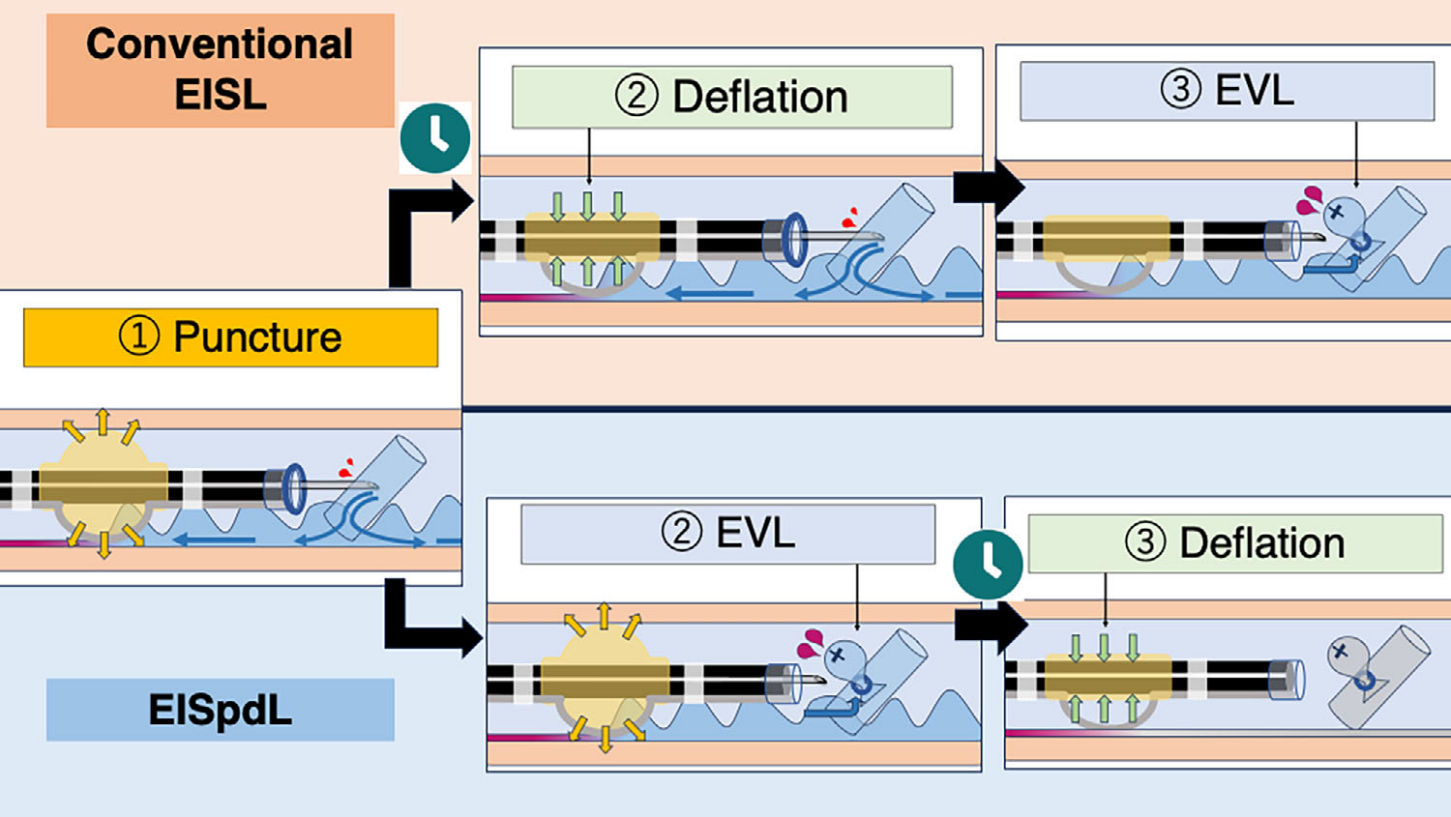
Comparison of the procedural steps between conventional endoscopic injection sclerotherapy with ligation (EISL) and EIS with pre‐deflation ligation (EISpdL). In conventional EISL, the varix is punctured, and the sclerosant is injected; after a retention period, the balloon is deflated, and endoscopic variceal ligation (EVL) is then performed at the puncture site for hemostasis. In EISpdL, the needle is withdrawn, and the puncture site is ligated by EVL while the balloon remains inflated, allowing stable sclerosant retention under continuous balloon compression and ensuring sufficient retention time.

We describe a modified EISL technique, EISpdL, in which the puncture site is ligated by EVL before the endoscope balloon is deflated. The technique was feasible in this case and required no additional devices or skills beyond those used in conventional EISL. A schematic comparison of the two techniques is shown in Figure 2.

The rationale for EISpdL is twofold. First, in the conventional sequence, EVL is applied only after balloon deflation; in the interval between deflation and ligation, blood flow is restored through a puncture site that is not yet closed, which may wash out the retained sclerosant and cause bleeding. Completing EVL before deflation eliminates this vulnerable interval. Second, and more importantly, maintaining balloon compression of the oral‐side variceal segment throughout the retention period sustains a static intravariceal sclerosant column, thereby prolonging contact between EO and the venous endothelium.

EVL alone might be considered sufficient, since it mechanically closes the puncture site. However, a single ligation point does not isolate a static sclerosant column along the entire treated segment and its afferent LGV, and afferent or collateral inflow can displace the sclerosant once oral‐side occlusion is released; in our experience, retention by EVL alone is often incomplete. Sustained balloon compression maintains stasis until adequate endothelial exposure is achieved. This is mechanistically relevant because EO‐induced thrombosis depends on both local concentration and exposure time, and experimental data indicate that longer stasis yields more complete thrombus formation [4, 8].

We combined EISpdL with gel‐immersion EIS, which has been reported to provide a high puncture success rate and frequent afferent‐vessel opacification and is used as the standard sclerotherapy technique at our institution [5, 6, 7]. Injecting the sclerosant into the afferent vessel under gel immersion, followed by pre‐deflation ligation, may act synergistically to improve treatment outcomes.

EISpdL is applicable to the same patient population as conventional EISL. It may be particularly useful in patients in whom stable needle positioning is difficult to maintain because of vigorous esophageal peristalsis or body movement, in whom the needle may otherwise become dislodged during injection. A limitation of the technique is that, because the needle is withdrawn before balloon deflation, additional sclerosant cannot be injected during the retention period; however, in most cases, nearly the full intended volume of EO can be delivered before ligation, which limits the practical impact of this constraint.

This report has important limitations. It describes a single case and demonstrates technical feasibility only. Although fluoroscopy showed retention of the sclerosant during balloon inflation and washout after deflation, this observation alone does not establish that retention is superior to that achieved with conventional EISL. Comparative data on retention time, thrombosis, technical success, and clinical outcomes were not obtained, and the purported advantages of the technique therefore remain to be confirmed. In addition, endoscopic ultrasonography (EUS) was not performed in this case. At our institution, pretreatment hemodynamic assessment is routinely performed with contrast‐enhanced computed tomography, and EUS is not performed routinely unless the computed tomography findings suggest a major portosystemic shunt or other features that require further hemodynamic evaluation; this was not the case in the present patient. Nevertheless, EUS can provide valuable information on variceal hemodynamics and anatomical characteristics that may help determine the indication for and strategy of sclerotherapy, and the absence of EUS assessment is a limitation of this report. Prospective studies accumulating additional cases and directly comparing EISpdL with conventional EISL are warranted.

In conclusion, EISpdL is a simple modification of conventional EISL that may enable more stable and prolonged intravariceal sclerosant retention. This case supports its technical feasibility and provides the basis for further comparative evaluation.

## Funding

The authors have nothing to report.

## Ethics Statement

This case report did not require institutional review board approval. The study was conducted in accordance with the Declaration of Helsinki.

## Consent

Written informed consent was obtained from the patient for the procedure and for the publication of this case report, including the accompanying images and video.

## Conflicts of Interest

The authors declare no conflicts of interest.

## Supporting information




**Supporting File 1**: deo270410‐sup‐0001‐VideoS1.mp4

## Data Availability

The data that support the findings of this study are available from the corresponding author upon reasonable request.
